# Application of Artificial Neural Networks for Producing an Estimation of High-Density Polyethylene

**DOI:** 10.3390/polym12102319

**Published:** 2020-10-10

**Authors:** Akbar Maleki, Mostafa Safdari Shadloo, Amin Rahmat

**Affiliations:** 1 Department for Management of Science and Technology Development, Ton Duc Thang University, Ho Chi Minh City, Vietnam; akbarmaleki@tdtu.edu.vn; 2 Faculty of Applied Sciences, Ton Duc Thang University, Ho Chi Minh City, Vietnam; 3 CORIA-UMR 6614, CNRS-University & INSA of Rouen, Normandie University, 76000 Rouen, France; 4 Institute of Research and Development, Duy Tan University, Da Nang 550000, Vietnam; 5 Faculty of Electrical—Electronic Engineering, Duy Tan University, Da Nang 550000, Vietnam; 6 School of Chemical Engineering, University of Birmingham, Birmingham B15 2TT, UK; a.rahmat@bham.ac.uk

**Keywords:** polyethylene, ethylene index, intelligence approaches, prediction

## Abstract

Polyethylene as a thermoplastic has received the uppermost popularity in a vast variety of applied contexts. Polyethylene is produced by several commercially obtainable technologies. Since Ziegler–Natta catalysts generate polyolefin with broad molecular weight and copolymer composition distributions, this type of model was utilized to simulate the polymerization procedure. The EIX (ethylene index) is the critical controlling variable that indicates product characteristics. Since it is difficult to measure the EIX, estimation is a problem causing the greatest challenges in the applicability of production. To resolve such problems, ANNs (artificial neural networks) are utilized in the present paper to predict the EIX from some simply computed variables of the system. In fact, the EIX is calculated as a function of pressure, ethylene flow, hydrogen flow, 1-butane flow, catalyst flow, and TEA (triethylaluminium) flow. The estimation was accomplished via the Multi-Layer Perceptron, Radial Basis, Cascade Feed-forward, and Generalized Regression Neural Networks. According to the results, the superior performance of the Multi-Layer Perceptron model than other ANN models was clearly demonstrated. Based on our findings, this model can predict production levels with R^2^ (regression coefficient), MSE (mean square error), AARD% (average absolute relative deviation percent), and RMSE (root mean square error) of, respectively, 0.89413, 0.02217, 0.4213, and 0.1489.

## 1. Introduction

Polyethylene as a thermoplastic has received the uppermost popularity in a vast variety of applied contexts. In spite of the need for considerable financial investment for producing polyethylene, the end consumer might receive a very inexpensive and throwaway product. Therefore, improvements in polymer fabrication procedures aiming at reducing manufacturing expenses remain as an investigational, developmental, and process expansion topic [1].

High-quality, low-cost petrochemical products are increasingly in demand for many applications including modernistic HDPE (high-density polyethylene) marketplaces [2]. Polyethylene is manufactured using common process technologies including high-pressure autoclave, high-pressure tubular, slurry (suspension), gas phase, and solution. The gas phase process in particular is one of the typical and frequently used technologies in the production of HDPE and other polyolefins. Various reactors are applied in ethylene polymerizations ranging from simple autoclaves and steel piping to CSTR (continuously stirred tank reactors) and vertical fluidized beds [3]. The main incentives for this innovation include the elimination of the necessity for removing the catalyst following the reaction and making the product in a style appropriate for manipulation and reposition. In the gas phase reactors, polymerization occurs at the surface of the catalyst and the polymer matrix, which is inflated with monomers throughout the polymerization [4].

Although catalysts for polymerizing ethylene are often heterogeneous, homogeneous catalysts are also used in some processes. Currently, four kinds of catalysts exist for polymerizing ethylene: Ziegler–Natta, Phillips, metallocenes, and late transition metal catalysts [2]. Only the first three types have commercial applications while the last one is yet to undergo examination and a developing phase. Ziegler–Natta catalysts vary greatly but are usually TiCl_4_ supported on MgCl_2_ and commonly employed for the polyethylene production in the industry [5].

In HDPE production practices, the ethylene index (EIX) is the critical controlling variable that indicates product characteristics. It is necessary to preserve the uniform features of HDPE throughout grade change practices to fulfill the different and strict requirements for HDPE products, including the EIX.

There are several approaches regarding polyethylene production estimation and correlation in the literature. Khare et al. [6] introduced steady-state and dynamic models for the HDPE slurry polymerization procedure for industrial applications. The authors utilized parallel reactors data to model the reactors in series order. They set the kinetic parameters by initial consideration of a single site catalyst followed by using the outcomes to optimize the kinetic parameters for multi-site-type catalyst. Neto et al. [7] designed a dynamic model for the LLDPE polymerization process on the basis of a multi-site catalyst that underwent development merely for a single slurry reactor. 

Considering a myriad of studies for improving polyethylene productivity, modeling approaches stand superior to experiments due to several key factors, including time and running costs, test feasibility, and safety (without hazards) concerns [8,9,10,11,12]. As denoted in previous publications, modeling approaches comprise of regression models, mathematical models, and artificial intelligence models. Nevertheless, the accurate function of mathematical models is dubious, in particular, once these models deal with very uncertain polyethylene manufacture. Regression models, however, can still well predict the means of production systems per month. As it is necessary to accurately model the productive status of such systems, artificial neural network (ANN) models have been used to achieve this objective due to their ability to handle great uncertainty of such data. ANNs integrate industrial data for predicting the rate of production, but only a few investigations have been successful in utilizing the ANN models for such systems by integrating a diverse range of techniques such as the MLPNN (multi-layer perceptron), CFNN (cascaded forward), RBFNN (radial basis function), and GRNN (general regression) neural networks [13,14], among others. 

MLP is a neural model with the highest prediction applicability, consisting of multiple layers, namely input, hidden layer(s), and output. Hidden layer(s) can also be set more precisely as having layers of nodes [15]. The MLPNN as the prime and plainest topology of the ANN, employed for updating the weight links through the learning step. 

RBFNN is a common feedforward neural network, which has been demonstrated to be capable of global estimation without local minima problem. Besides, it possesses a plain construct and a rapid learning algorithm in comparison with other neural networks [16]. Despite the availability of multiple activation functions for radial basis neurons, the Gaussian function has received the uppermost popularity. Training is necessary before applying an RBF ANN, which is commonly achievable in two stages. Choosing centers among the data applied is in the first stage of the training. The second stage is to utilize the normal least squares for linear estimation of one weighting vector. The self-organized center election is the widespread learning approach employed for selecting RBF centers.

The input and output neurons begin the production of the CFNN topology. The output neurons are present in the neural network in advance; thus, novel neurons are provided for the network, as a result of which the network, in turn, attempts to increase the correlation level between the outputs and inputs by comparing the network residue with the novel measured error. This procedure goes ahead until reaching a smaller error value in the network, which explains the reasoning that it is labeled as a cascade [17]. CFNN generally comprises three major layers namely, input, hidden, and output layers. The variables in hidden layers are multiplied by the bias (1.0) and the weight (computed in the creation phase to decline the prediction error) followed by addition to the sum entering the neuron. The resultant value from this procedure will cross a transfer function to present the output value. 

GRNN is a type of supervised network that works on the basis of the probabilistic model and is able to produce continuous outputs. It is a robust instrument for non-linear regression analysis based on the approximation of probability density functions using the Parzen window technique [18]. The GRNN architecture basically does not need an iterative process to simulate such results as back-propagation learning algorithms. GRNN is capable of estimating arbitrary functions among output and input datasets directly from training data.

The main uses of the RBF and GRNN topologies are with a rather small size of input data. Despite the common topology of neural networks, every neuron depends on the entire prior layer neurons in the CFNN. Additionally, the CFNN is able to carry on to a broad extent in case the input data possess a sizable memory capacity.

As revealed by a literature review, the ANN model has applications in predicting the performance of production rate. Nonetheless, only the MLP model was utilized to forecast the production rate [19]. The novelty of the present study is to utilize and compare the performance of several models including the GRNN and RBF models, which were not previously used for predicting the production rate. Accordingly, the current research mainly aims to introduce and assess a model for predicting the rate of polyethylene fabrication. The novelty of our model is characterized by its capability in predicting system productivity by taking the uncertainty issue into account. 

## 2. Methods

### 2.1. HDPE Process

**Figure 1:**
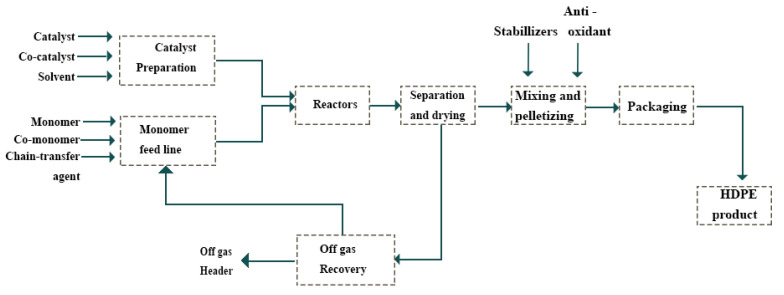
A diagram of slurry polymerization for HDPE production [20].

The HDPE plant comprises two procedures, through which the polymerization reaction is put into action. Figure 1 illustrates a representative gas phase polymerization procedure for producing HDPE introduced by this research. Every process involves two polymerization reactors. The polymerization reaction was very exothermal, with a heat reaction of around 1000 kcal/Kg of ethylene.

There is, therefore, a need to provide suitable cooling systems that eliminate nearby 80% of heat in the polymerization process. Co-monomer (including 1-butene or higher alpha-olefin), ethylene, an activator, hydrogen, hexane, and a catalyst, as well as continually recycled original liquid, are supplied to reactors as reactants. Generally, the slurry phase occupies almost 90–95% of reactor volume. With building up of the reaction pressure, the polyethylene slurry is transferred to the next process apparatus and the reactor level is preserved within an allowable range. Separating the reaction slurry in the centrifugal separator yields cake, holding dilutants, after which dilutants are removed with hot nitrogen gas in a dryer. Thereafter, suitable additives are added depending on the final usages. After pelletizing in water, pellets are dried and placed in a homogenizer followed by cooling.

### 2.2. Artificial Neural Networks

The ANN is an AI (artificial intelligence) method, defined as the information processing model, which is exhilarated by the human nervous systems for information processing [21,22,23]. The ANN is capable of identifying patterns and learning from their interplays with the environment [24,25,26,27,28]. An ANN is constructed through three major fractions of the input, the output, and the hidden layer(s), all of which comprise parallel units named neurons [29]. The neurons are coupled with massive weight links, allowing the information to be transformed among the layers. The ANN model is essentially dependent upon two key steps for predicting the response of different systems: the training phase and the testing phase. In the training phase, the inputs are received by the neurons over their entering connections, after which these inputs are combined by a specific action with the output to discover the best the weight links values. Therefore, records of the association between output and input variables are taken to forecast the fresh data. In the testing phase, the system performance is tested using a portion of the input data and a comparison is made between the predicted data and the real data. The principal benefit of the ANN model is its ability in solving sophisticated problems that cannot be easily solved by traditional models; it is also capable of solving problems without an algorithmic solution or those with algorithmic solutions having sophisticated definitions.

**Figure 2:**
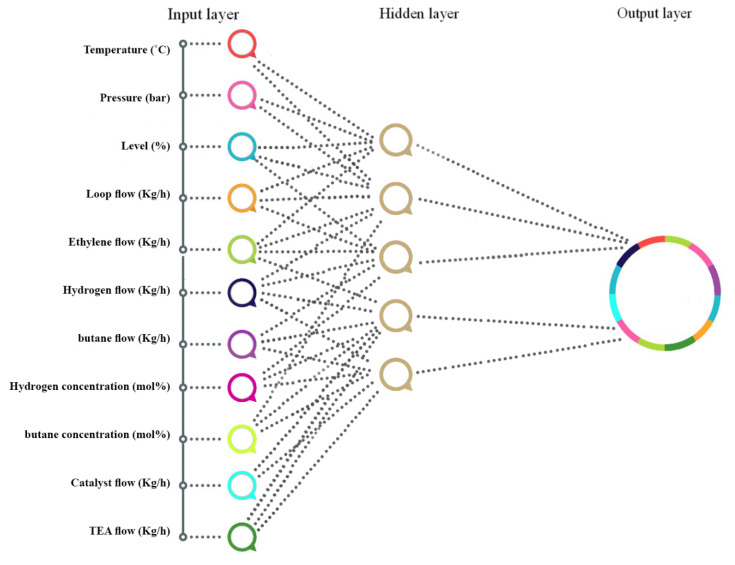
Schematic representation of the used model for neural networks [30].

The independent variables from outside resources in the input layer are processed by several mathematical operators and by sending values to the hidden layers. On the other hand, output neuron(s) are used to determine the dependent variables. The output value for all ANN structures can be defined as below:

(1) $$Output=f\left(\overset{n}{\underset{i=1}{\sum }}w_{i}x_{i}+b\right)$$

where this value is computed by the activation function of f. Here, b is the summation of the bias value, and $w_{i}$ is the weight of $x_{i}$ input. Figure 2 presents the schematic diagram of the proposed neural network for simulating the EIX.

### 2.3. Accuracy Assessment of AI Models

The current study has designed various AI-based approaches with diverse topologies to opt for the best model based on the accuracy of predictability. This can serve as a selection paradigm for network configuration to determine the number of hidden layers and neurons, the spread factor, and training algorithm. It is noted that the dependencies of the neurons number in the hidden layer on prediction intervals were also examined. The performance of ANN models using R^2^ (regression coefficient), MSE (mean square error), AARD% (average absolute relative deviation percent), and RMSE (root mean square error) are calculated, respectively, as the following:

(2) $$MSE\;=\;\frac{1}{N}\;{\sum_{i=1}^{N}\left(Y_{i,act}-Y_{i,pred}\right)}^{2}$$

(3) $$RMSE\;=\;\sqrt{\frac{1}{N}\;{\sum_{i=1}^{N}\left(Y_{i,act}-Y_{i,pred}\right)}^{2}}$$

(4) $$AARD\%=\frac{1}{N}\sum_{i=1}^{N}\left(\left|\frac{Y_{i,act}-Y_{i,pred}}{Y_{i,act}}\right|\right)\times 100$$

(5) $$R^{2}=\frac{\sum_{i=1}^{N}{\left(Y_{i,act}-{\bar{Y}}_{act}\right)}^{2}-\sum_{i=1}^{N}{\left(Y_{i,act}-Y_{i,pred}\right)}^{2}}{\sum_{i=1}^{N}{\left(Y_{i,act}-{\bar{Y}}_{act}\right)}^{2}}$$

where, $Y_{i,\;act}$ is the actual and $Y_{i,\;pred}$ is predicted value. *N* and ${\bar{Y}}_{act}$ also denote the number of data points and the mean of actual values, respectively.

To evaluate mean squared error (MSE) for various parameters, the predicted data are also subject to statistical analysis. MSE measures the absolute deviation of the predicted and the actual values. Positive and negative values denote overestimation and underestimation of parameters, respectively. Based on the aforesaid description, the RMSE denotes that the model is efficient on the basis of the difference between the predicted and real data. Accordingly, a large positive RMSE indicates the presence of a high deviation between the predicted and real data and in a reverse order. The R^2^ index defines the proximity of the actual data points to the predicted values. 

## 3. Results and Discussion

This section summarizes the actual databank gathered from the industrial polyethylene petrochemical company, by considering the significant independent variables and by utilizing the Pearson correlation matrix. Furthermore, this section deals with determining the best structures of different models and comparing the precisions of different models. The present section concludes by selecting the best model and analyzing the results.

### 3.1. Industrial Database

To investigate the EIX, eleven independent sets of input data namely, temperature, operating pressure, level, loop flow, ethylene flow, hydrogen flow, 1-butane flow, hydrogen concentration, 1-butane concentration, catalyst flow, and TEA (triethylaluminium) flow (i.e., inputs 1 to 11 denoted, respectively, by X1 to X11), and the EIX response (denoted by Y) are collected. Table 1 presents the information summary of the industrial data used in this work.

According to the industrial databank, 93 data points were gathered at the steady-state conditions. The trained neural network needs validation for determining the precision of the introduced model. The network performance can be analyzed by cross-validation of an unidentified dataset. The network is validated through preserving a fraction of the dataset (e.g., 15%) for validation and the rest of the dataset is used for training. After the training phase, the data forecasted through the ANN topology and the measured data undergo a correlation analysis. 

All ANN models were developed in MATLAB^®^ with the Levenberg–Marquardt optimization algorithm. Besides, the choice of training algorithm and neuron transfer function has a major contribution to model precision. As researchers have shown, the Levenberg–Marquardt (LM) algorithm produces quicker responses for regression-type problems in overall facets of neural networks [31,32]. Most often, the LM training algorithm was reported to have the highest significant efficiency, fast convergence, and accuracy compared with other training algorithms.

### 3.2. Scaling the Data 

To enhance the rate of convergence in the training step as well as to avoid parameter saturation in the intended ANNs, the entire actual data were subjected to mapping within the interval [0.01 0.99]. Data were normalized by Equation (6):

(6) $$V_{normal}\;=0.01\;+\;\frac{V\;-\;V_{\min}}{V_{\max}\;-\;V_{\min}}\;\times \;\left(0.99\;-\;0.01\right)$$

where V denotes an independent or dependent variable, $V_{normal}$ represents the normal value, $V_{max}$ is the maximum, and $V_{min}$ is the minimum value of each variable.

### 3.3. Independent Variable Selection

**Figure 3:**
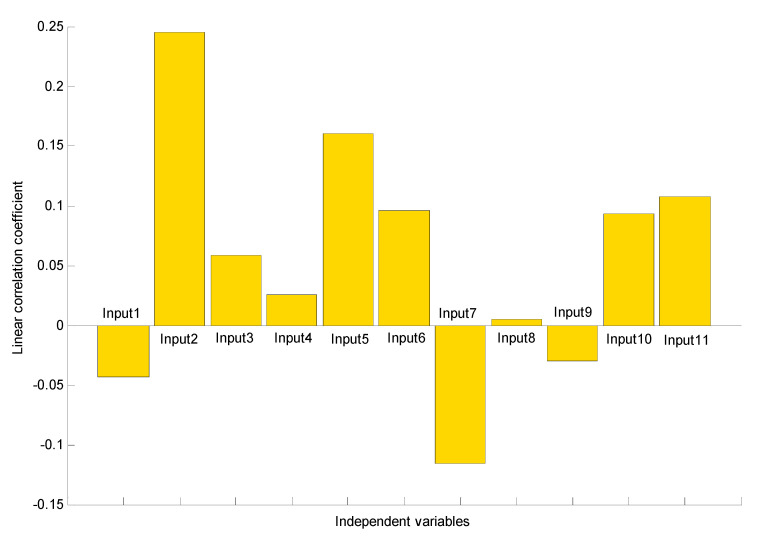
Pearson correlation coefficient values among independent and dependent variables.

Mathematical investigation for the dependency of two variables is possible through the correlation matrix study, the coefficients of which are usually measured from −1 to +1. This indicates that the two variables are correlated directly or indirectly given the signs of these coefficients, whereas the magnitude defines the robustness of their association. Our research surveyed a multivariate AI-based method with the Pearson correlation test for estimating the degree of relationship between each two variables [23]. Figure 3 displays the correlation coefficient values’ given probable pairs of variables.

The Pearson correlation coefficient as the variable ranking is described in choosing appropriate inputs for the neural network [33,34]. Values delivered by the Pearson method reveal the type and intensity of the association between every variable pair, with a value between −1 and +1 representing the uppermost converse relationship and the highest direct association, respectively. The coefficient takes a zero value in cases where the given variables do not have any association. Independent variables take non-zero correlation coefficients that verify their choices. The highest consideration is devoted to absolute average values because they have important strong associations. The values of Pearson correlation coefficient for each input are presented in Table 2.

Accordingly, this examination confirmed that Input2, Input5, Input6, Input7, Input10, and Input11 had the uppermost direct dependency and that other inputs presented the lowermost indirect association. Hence, it is possible to model polyethylene as a function of pressure, ethylene flow, hydrogen flow, 1-butane, catalyst flow, and TEA flow. Therefore, we try to present a smart model to derive the following relation:

(7) Output= function (Input 2, Input 5, Input 6, Input 7, Input 10, Input 11)

Consequently, in the case of maximizing the AAPC (average of absolute Pearson’s coefficient) for a specific transformation on the dependent variable, it is inferred that it yields an association between the dependent and independent variables with the highest reliability. Every input variable of differing models was selected with the Pearson correlation coefficient. The inputs of every model are presented in Table 3. 

From this table, it can be concluded that it is better to output to the power of 12 instead of modeling the output itself. Although, at last, by inverse transformation, the dependent variable is calculated to compare with the actual values.

### 3.4. Configuration Selection for Different ANN Approaches

In this work, the EIX is predicted using a proper ANN model obtained from a logical procedure. As mentioned earlier, the number of hidden neurons has a major contribution to network performance. The majority of related investigations obtain the number of neurons through the trial and error approach. Training and generalization errors may highly happen when the numbers of hidden neurons are less than the optimum numbers. On the other hand, larger numbers of hidden neurons may result in over-fitting and considerable variations. Therefore, it is necessary to calculate the optimum number of hidden neurons for achieving the best performance of the network.

Subsequently, the ANN approaches were developed and then, for example, the MLP network was compared in terms of performance with CF, RBF, and GR neural networks. The numerical validation associates with the observed AARD%, R^2^, MSE, and RMSE between actual and estimated data. According to the literature, MLP network capability with one hidden layer was proven [35]. As such, an MLP network with only a single hidden layer is used for the analysis.

It is noted that the training data points should be at least twice the number of bias and weights. As a result, for the MLP with one dependent and six independent variables, the hidden neuron is computed as:

(8) 2× (8N+1)≤ 64 (training data points) N≤4

Therefore, this number can change from 1 to 4 (the highest acceptable number) in this network, and is trained 50 times for each network. The best configuration of the hidden neurons in the MLP model is presented in Table 4.

**Figure 4:**
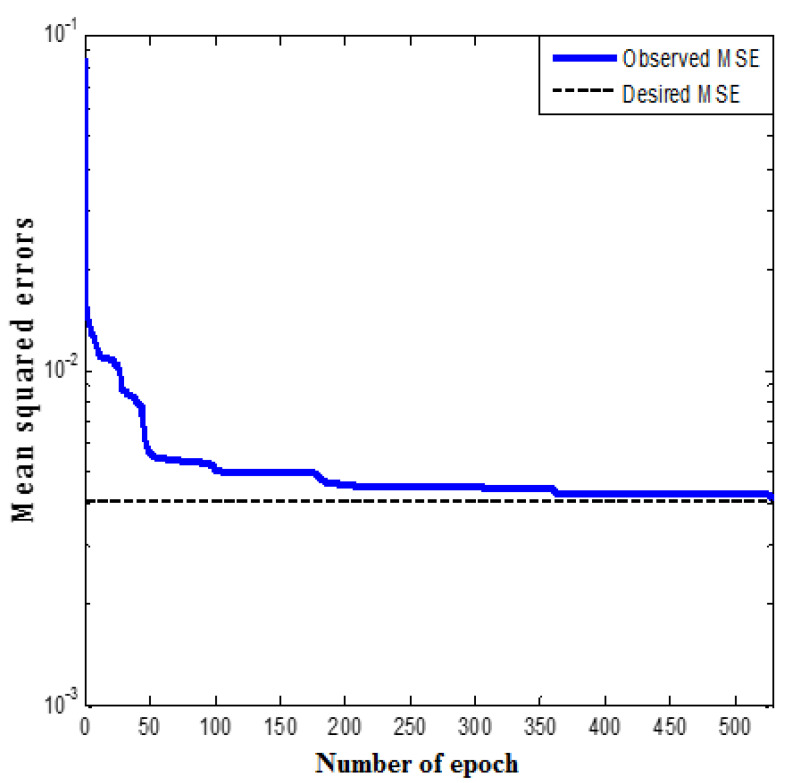
The MSE for the multi-layer perceptron (MLP) network with 1 to 4 hidden neurons (50 networks per neuron) during the training stage.

The MLP network with three hidden neurons and the structure of 6-3-1 was determined as the most appropriate model. The MSE values of the MLP network with various numbers of hidden layer neurons are presented in Figure 4. The data reveal that the optimality of the three numbers of neurons owes to the uppermost value of R^2^ (0.89413) and the lowermost value of MSE (0.02217).

**Figure 5:**
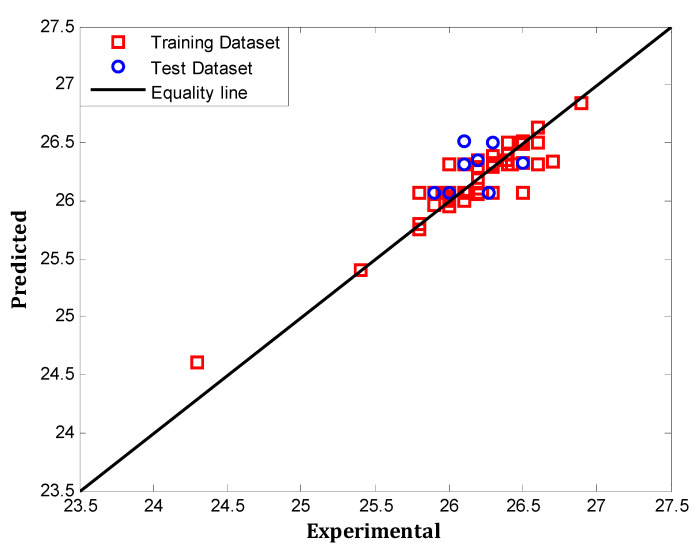
The efficiency of the optimum MLP model for prediction of all the datasets.

According to Figure 4, MSE is lowest (0.07184) in the total MLP model with the presence of a single neuron in the hidden layer. The MLP model possesses the least MSE (0.02217) once six neurons exist in the hidden layer. Moreover, Figure 5 presents a comparison between the industrial datasets and the predicted values by the optimum MLP network. The fit performance was determined for every trained MLP with minimum MSE value on the basis of R^2^ values.

### 3.5. Other Types of ANN

To find an appropriate model for evaluating the EIX, different topologies of artificial neural networks must be compare based on their performances. Therefore, the intended MLP approach developed with optimum configuration in terms of predictive accuracy was evaluated with other ANN models (GR, CF, and RBF). The sensitivity results for selecting the best number of hidden neurons are presented in Table 5, Table 6 and Table 7. It should be pointed out that determination of the number of hidden neurons in the other ANNs was the same as for the MLP model.

In GR, hidden neurons were not significant and the spread value needed to be set up. Subsequently, the spread value for the GR changes from 0.1 to 10 with 0.1 steps and 50 different GRs were considered, with statistical indices. The MSE is minimum (0.10808) in the GR model when the spread value was 4.81. 

Considering the task, the best model contains the lowest value for MSE and AARD%. Table 8 clearly reveals that the MLP model can predict the EIX more accurately than other types of ANN models. Based on the statistical error values, the MSE for the MLP model (0.02217) is less than those for the MSE estimated with CF (0.03914), GR (0.10808), and RBF (0.09255) models, respectively. The above findings confirm that the MLP model is superior in the prediction of the EIX comparing to other ANN models. 

The MLP model, trained by the Levenberg–Marquardt algorithm with 6-3-1 structure, has the *logsig* transfer function in the output and hidden layers. In fact, this model is chosen from 600 models (200 MLPNN models, 150 CFNN models, 50 GRNN models, and 200 RBFNN models). This selection is based on four statistical indices: AARD%, MSE, RMSE, and R^2^.

Table 9 summarizes the value of the weight and bias for the proposed MLP model. The MLP was trained using a training dataset by the adjustment of the biases and weights. The performance validity of the trained MLP was achieved according to the training and testing datasets (independent datasets). The optimal division ratio is 85:15 for segregating the data.

## 4. Procedure for Simple Usage of the MLP Model

1. All independent variables normalize into an interval of [0.01 0.99] using Equation (9) and should be arranged as a 6×1 vector.

(9) $$V_{normal}\;=0.01\;+\;\frac{V\;-\;V_{\min}}{V_{\max}\;-\;V_{\min}}\;\times \;\left(0.99\;-\;0.01\right)$$

2. Multiply the first six columns of Table 9 by the variables achieved in step 1.
3. The 7th column of Table 9 is added to the obtained values in step 2.
4. Substitute all elements of step 3 in the following equation to calculate *NO_HL_*.

*NO_HL_* = 1/[1 + exp(−values obtained in step 3)] (10)

5. Multiply the transposition of the 8th column of Table 9 by the obtained values in step 4.
6. Add the value of the last column of Table 9, i.e., 0.69286, to the obtained values in step 5.
7. Substitute the obtained value in step 6 in the following equation to calculate *NO_OL_*.

*NO_OL_* = 1/[1 + exp(−value obtained in step 6)] (11)

8. Inverse transformation using NO_OL_ ^ (1/12).
9. Map the output values in the previous step into the actual range of dependent variables, i.e., [24.3 26.9], using the following equation.

Predicted variable = (Step9 − 0.01) × 2.6531 + 24.3(12)

10. The obtained value in step 9 shows the estimated value for the dependent variable by the proposed MLP approach.

**Figure 6:**
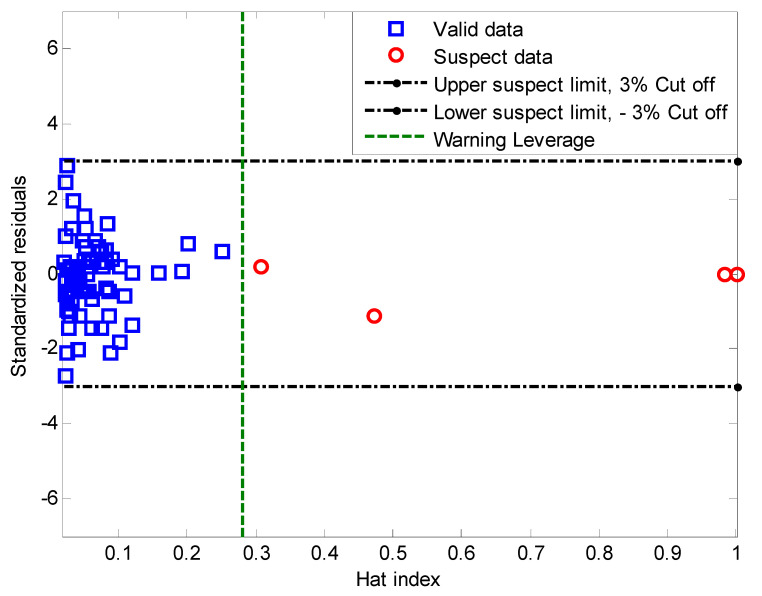
Williams plot for the MLP model.

Finally, the proposed MLP model was statistically checked for reliability, so the leverage approach was used. The following figure shows the outlier detection based on the MLP model using the Williams Plot method. It is clear from Figure 6 that only 4 data points (red dots) of the 75 data available are difficult to model. In fact, about 95% of the data is in the valid range (blue squares).

## 5. Conclusions

In HDPE procedures, the EIX is the critical controlling variable indicative of product quality. A large number of approaches are available for the estimation and correlation of EIX, since it is difficult to nonlinearly measure them. The current paper applied predicting methods to prediction schemes, including MLPNN (multi-layer perceptron), CFNN (cascaded forward), RBFNN (radial basis function), and GRNN (general regression) neural networks. Comparisons were made between the findings of different dynamic prediction schemes to assess the best performance. The superior performance of the present MLP model was demonstrated using the same case study dataset for predicting the EIX than other models. The results clearly suggest that three hidden neurons are the best number of neurons in the proposed MLP model. For this model, the MSE and R^2^ values of the total dataset are 0.02217 and 0.89413, respectively. The main advantages of using ANNs for the EIX are the ability to predict the production rate of the network quickly and to clarify the characteristics of high-density polyethylene with network inputs. Although these models have used complex computational algorithms, fast convergence along with accuracy is not always confirmed in some cases.

## Figures and Tables

**Table 1:** Ranges and of the Industrial inputs.

Independent Variables	Named	Minimum	Maximum
Temperature (°C)	Input 1	77	97.8
Pressure (bar)	Input 2	21.8	22.6
Level (%)	Input 3	68	77
Loop flow (Kg/h)	Input 4	646	724
Ethylene flow (Kg/h)	Input 5	1.8	15.6
Hydrogen flow (Kg/h)	Input 6	6.1	25
1-butane flow (Kg/h)	Input 7	17	970
Hydrogen concentration (mol%)	Input 8	16.02	19.03
1-butane concentration (mol%)	Input 9	0.49	1.38
Catalyst flow (Kg/h)	Input 10	2	3.7
TEA flow (Kg/h)	Input 11	1.8	3.7

**Table 2:** The Pearson correlation coefficient for each input.

Independent Variables	Pearson Correlation Coefficient
Input1	−0.0430
Input2	0.2452
Input3	0.0581
Input4	0.0252
Input5	0.1606
Input6	0.0956
Input7	−0.1160
Input8	0.0052
Input9	−0.0296
Input10	0.0935
Input11	0.1075

**Table 3:** Pearson coefficient values calculated for different transformations between dependent and independent variables.

Transformation	Pearson’s Coefficient						AAPC
	Input11	Input10	Input7	Input6	Input5	Input2	
Output^15^	0.09837	0.08478	−0.12881	0.0877	0.17071	0.28188	0.14204
Output^14^	0.09943	0.08576	−0.12845	0.08885	0.17077	0.27997	0.1422
Output^13^	0.10045	0.0867	−0.12801	0.08992	0.17073	0.27795	0.14229
**Output^12^**	**0.10143**	**0.0876**	**−0.12750**	**0.09092**	**0.17058**	**0.27584**	**0.14231**
Output^11^	0.10235	0.08845	−0.12690	0.09184	0.17033	0.27361	0.14225
Output^10^	0.10321	0.08925	−0.12623	0.09268	0.16996	0.27128	0.1421
Output^2^	0.1074	0.09331	−0.11754	0.09572	0.16225	0.24851	0.13745
Output	0.10751	0.09346	−0.11603	0.09556	0.16063	0.24516	0.13639
Output^0.75^	0.10753	0.09349	−0.11563	0.0955	0.1602	0.24431	0.13611
Output^0.5^	0.10753	0.0935	−0.11523	0.09543	0.15976	0.24344	0.13582
Output^0.25^	0.10753	0.09351	−0.11483	0.09535	0.15932	0.24258	0.13552
Output^0.1^	0.10753	0.09352	−0.11458	0.0953	0.15904	0.24205	0.13534
Output^−0.1^	−0.10752	−0.09352	0.11425	−0.09523	−0.15867	−0.24135	0.13509
Output^−0.25^	−0.10751	−0.09352	0.114	−0.09517	−0.15839	−0.24082	0.1349
Output^−0.5^	−0.10749	−0.09351	0.11357	−0.09507	−0.15791	−0.23994	0.13458
Output^−0.75^	−0.10747	−0.09350	0.11314	−0.09496	−0.15743	−0.23904	0.13426
Output^−1^	−0.10743	−0.09348	0.11271	−0.09484	−0.15693	−0.23814	0.13392
Output^−2^	−0.10723	−0.09335	0.11091	−0.09427	−0.15485	−0.23449	0.13252
Output^−10^	−0.10154	−0.08867	0.09353	−0.08469	−0.13296	−0.20259	0.11733
Output^−11^	−0.10034	−0.08765	0.09106	−0.08289	−0.12965	−0.19840	0.115
Output^−12^	−0.09903	−0.08653	0.08855	−0.08097	−0.12624	−0.19420	0.11259
Output^−13^	−0.09763	−0.08533	0.08599	−0.07894	−0.12273	−0.19000	0.11011
Output^−14^	−0.09614	−0.08405	0.08341	−0.07680	−0.11914	−0.18581	0.10756
Output^−15^	−0.09456	−0.08269	0.0808	−0.07457	−0.11548	−0.18165	0.10496
exp(Output)	0.08336	0.07114	−0.12809	0.07017	0.16382	0.29757	0.13569

**Table 4:** The procedure for detecting the best configuration for the MLP model.

Hidden Neuron	Dataset	Statistical Index			
		AARD%	MSE	RMSE	R^2^
1	Train	0.6566	0.07274	0.2697	0.6043
	Test	0.7578	0.06665	0.2582	0.35034
	Total	0.6715	0.07184	0.268	0.57942
2	Train	0.568	0.06603	0.257	0.66405
	Test	0.7314	0.05583	0.2363	0.65288
	Total	0.592	0.06454	0.254	0.644
**3**	**Train**	**0.3863**	**0.01982**	**0.1408**	**0.91723**
	**Test**	**0.6251**	**0.03581**	**0.1892**	**0.53962**
	**Total**	**0.4213**	**0.02217**	**0.1489**	**0.89413**
4	Train	0.3397	0.02037	0.1427	0.90878
	Test	0.8084	0.07617	0.276	0.44131
	Total	0.4085	0.02856	0.169	0.86377

**Table 5:** Determination of the best-hidden neurons for cascaded forward (CF).

Hidden Neuron	Dataset	Statistical Index			
		AARD%	MSE	RMSE	R^2^
1	Train	0.5604	0.07049	0.2655	0.62338
	Test	0.6377	0.05431	0.233	0.58962
	Total	0.5717	0.06812	0.261	0.60867
2	Train	0.4961	0.05227	0.2286	0.74815
	Test	0.6904	0.06022	0.2454	0.36408
	Total	0.5246	0.05344	0.2312	0.71356
**3**	**Train**	**0.488**	**0.03638**	**0.1907**	**0.83777**
	**Test**	**0.5729**	**0.0552**	**0.235**	**0.48687**
	**Total**	**0.5004**	**0.03914**	**0.1979**	**0.79892**

**Table 6:** Determination of the best hidden neurons for radial bias function (RBF).

Hidden Neuron	Spread	Dataset	Statistical Index			
			AARD%	MSE	RMSE	R^2^
1	0.41	Train	0.762	0.10257	0.3203	0.30383
		Test	0.8574	0.06878	0.2623	0.28658
		Total	0.776	0.09762	0.3124	0.312
**2**	**0.81**	**Train**	**0.7563**	**0.09887**	**0.3144**	**0.42263**
		**Test**	**0.762**	**0.05584**	**0.2363**	**−0.10195**
		**Total**	**0.7571**	**0.09255**	**0.3042**	**0.38919**
3	1.01	Train	0.7311	0.09259	0.3043	0.45482
		Test	0.774	0.05724	0.2392	0.33916
		Total	0.7374	0.08741	0.2956	0.43965
4	0.21	Train	0.6708	0.08324	0.2885	0.51622
		Test	0.7579	0.08244	0.2871	0.4649
		Total	0.6836	0.08312	0.2883	0.48421

**Table 7:** Sensitivity analyses on spread parameter for finding best hidden neurons for general regression (GR).

Spread	Dataset	Statistical Index			
		AARD%	MSE	RMSE	R^2^
4.81	Train	0.8361	0.10982	0.3314	0.32248
	Test	0.9242	0.098	0.313	−0.20907
	Total	0.849	0.10808	0.3288	0.22754

**Table 8:** A performance comparison of ANN models.

Model	Dataset	Statistical Index			
		AARD%	MSE	RMSE	R^2^
MLP	Train	0.3863	0.01982	0.1408	0.91723
	Test	0.6251	0.03581	0.1892	0.53962
	Total	0.4213	0.02217	0.1489	0.89413
CF	Train	0.488	0.03638	0.1907	0.83777
	Test	0.5729	0.0552	0.235	0.48687
	Total	0.5004	0.03914	0.1979	0.79892
GR	Train	0.8361	0.10982	0.3314	0.32248
	Test	0.9242	0.098	0.313	−0.20907
	Total	0.849	0.10808	0.3288	0.22754
RBF	Train	0.7563	0.09887	0.3144	0.42263
	Test	0.762	0.05584	0.2363	−0.10195
	Total	0.7571	0.09255	0.3042	0.38919

**Table 9:** Weight and bias coefficients for the proposed MLP model.

Weights between Hidden Layer Neurons and Input Variables						Bias of Hidden Layer Neurons	Weight between the Hidden Layer and the Output Layer	Output Layer Bias
Input2	Input5	Input6	Input7	Input10	Input11			
37.522	325.0769	−1007.47	−3.6761	−330.513	−168.349	176.6308	−0.94888	0.69286
272.4066	−70.6262	−94.8238	4.1889	−150.163	2.1714	−16.8291	2.7494	
−1095.55	1458.018	70.1148	370.1613	−539.429	−12.1413	9.7747	−2.276	

## Nomenclature

b	Bias
N	Number of actual data
X_i_	i^th^ input variable
Y	Response
w	Weight

## Abbreviations

AARD%	Average absolute relative deviation percent
AI	Artificial intelligence
ANN	Artificial neural networks
CFNN	Cascade feedforward neural networks
CSTR	Continuously stirred tank reactors
EIX	Ethylene index
HDPE	High-density polyethylene
MLP	Multi-layer perceptron
MLPNN	Multi-layer perceptron neural networks
MSE	Mean squared errors
RBF	Radial basis neural networks
RMSE	Root mean square errors
R2	Regression coefficient
TEA	Triethylaluminium

## Subscripts/Superscripts

pre.	Predicted variable
act.	Actual variable
max	Maximum value
min	Minimum value
normal	Normalized values
